# Successful Treatment of Mild Pediatric Kasabach-Merritt Phenomenon with Propranolol Monotherapy

**DOI:** 10.1155/2014/364693

**Published:** 2014-05-22

**Authors:** Worawut Choeyprasert, Rungrote Natesirinilkul, Pimlak Charoenkwan

**Affiliations:** Department of Pediatrics, Faculty of Medicine, Chiang Mai University, Chiang Mai 50200, Thailand

## Abstract

Kasabach-Merritt phenomenon (KMP) is relatively rare in childhood and adolescents with high mortality rate because of its hemorrhagic complications and unresponsiveness to treatments such as corticosteroids, vincristine, intravascular embolization, and/or surgery. Propranolol, a **β**-adrenergic receptor blocker, has a promising efficacy against vascular tumors such as infantile hemangiomas. But limited and variable data has been reported regarding the role of propranolol in treatment of KMP. We herein reported the successful treatment of mild pediatric KMP with propranolol monotherapy in a case of a five-week-old child with kaposiform hemangioendothelioma with successful treatment of both clinical and hematologic responses. After eight months of follow-up, patient still had stable cutaneous lesion while receiving propranolol monotherapy. Regular hematologic monitoring was done in order to detect any late relapse of the disease. Six months after discontinuation of propranolol, patient has still remained free of hematologic relapse, and primary cutaneous lesion has become a pale pink, 1 cm sized skin lesion.

## 1. Introduction

Kasabach-Merritt phenomenon (KMP) is characterized by a vascular tumor called kaposiform haemangioendothelioma (KHE), with evidence of thrombocytopenia, hypofibrinogenaemia, and/or coagulopathy. The condition was first described by Kasabach and Merritt in 1940 [1]. Despite the aggressive treatment of KMP, the mortality rate is still as high as 10–37% because of hemorrhagic complications and unresponsiveness to treatment [2–4]. Several treatment modalities of KMP, such as corticosteroids, vincristine, intravascular embolization, and surgery, have been described with variable responses. Controlled trials have not been conducted because of the relative rarity of the phenomenon and the lack of standard criteria for interpretation of treatment response.

Propranolol is a nonselective *β*-adrenergic receptor blocking agent that has a promising efficacy against vascular tumors such as infantile haemangiomas (IHs) [5–7]. In 2011, a case report described the effectiveness of oral propranolol in combination with four doses of weekly intravenous vincristine in treatment of KHE with KMP [8]. However, in a later report by Chiu et al. [9] in a case series of six KHE with KMP, only one out of four patients showed responsiveness (normalization of hematologic parameters of KMP) to propranolol used in combination with prednisolone, either with or without vincristine, while one out of two patients showed responsiveness to propranolol monotherapy.

We demonstrated the successful treatment of mild pediatric KMP with propranolol monotherapy in a case of a five-week-old child with successful treatment of both clinical and hematologic responses. After eight months of follow-up, patient still had stable cutaneous lesion while receiving propranolol monotherapy. Regular hematologic monitoring was done in order to detect any late relapse of the disease. After discontinuation of propranolol, patient has still remained free of hematologic relapse, and primary cutaneous lesion has become a pale pink, 1 cm sized skin lesion.

## 2. Case Presentation

A five-week-old female infant was admitted to our hospital due to productive cough with blood-stained sputum one day prior to hospitalization. She had been observed as having a violaceous, indurated mass at her left temporal area since birth, sized 0.5 cm × 0.5 cm, with close follow-up from that time onward. At four weeks of age, she developed progressive enlargement of the mass, without pain, to a size of 4 cm × 4 cm. At five weeks of age, she developed a frequent cough with one episode of blood-stained sputum.

**Figure 1:**
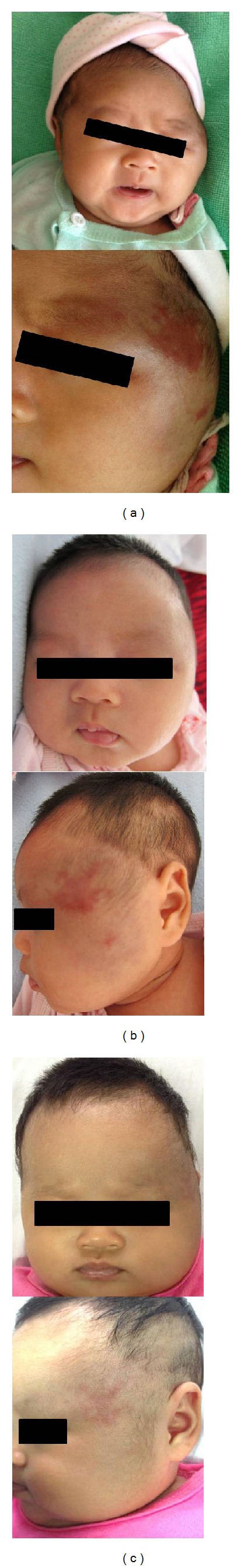
(a) Showing the patient at 5 weeks of age, before treatment with propranolol (at a dose of 2 mg per kilogram of body weight per day). (b) Showing the patient at 3 weeks after the initiation of propranolol with some degree of regression of primary lesion. (c) Showing the patient at 9 months of age (8 months after the initiation of propranolol treatment) with regression of primary lesion.

General physical examination was otherwise normal without dyspnea except for a pink purplish indurated mass, sized 5 cm × 5 cm, on left temporal area involving lateral part of eyelids, as shown in Figure 1. Ophthalmologic examination was normal. Direct laryngoscopy revealed no abnormal vessels or mass over oropharynx and vocal cords.

Initial laboratory values showed a hemoglobin level (Hb) of 9.9 g/dL, thrombocytopenia (97,000/*μ*L), and evidence of coagulopathy (a fibrinogen level of 113 mg/dL, normal 162–378 mg/dL; a D-dimer plasma level of 7.28 *μ*g/mL, normal 0.11–0.42 *μ*g/mL; prothrombin time (PT) of 11.2 sec, normal 10–14.3 sec, INR (international normalized ratio) of 1.06; activated partial thromboplastin time (APTT) of 38.80 sec, normal 32–55.2 sec). There was no evidence of microangiopathic hemolytic anemia (MAHA) in peripheral blood smear.

The diagnosis of KMP was made on the second day of admission based on the presence of hypervascularized mass, thrombocytopenia, elevated D-dimer, and hypofibrinogenemia without evidence of apparent infection. Computerized tomography (CT) and CT angiography (CTA) of the head and neck revealed an arterial-enhancing intermediate attenuated soft-tissue lesion with prominent size of internal vessels involving left hemifacial region without abnormal calcification, compatible with KHE. No biopsy was done to confirm the diagnosis of KHE due to high risk of developing procedure-related complications including bleeding. Platelet count decreased to 55,600/uL without episodes of bleeding. The patient was stable.

**Figure 2:**
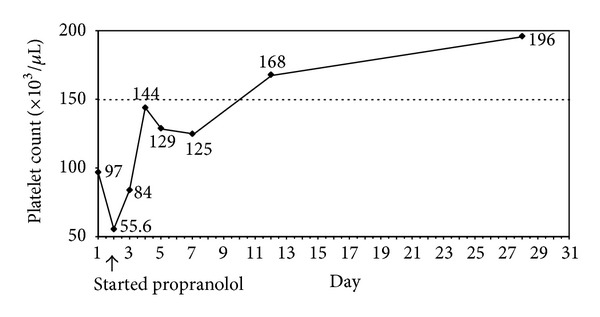
Course of treatment and sequential platelet count.

Due to stable clinical status and mild derangement of hematologic parameters (platelets > 50,000/uL and fibrinogen > 100 mg/dL), we started oral propranolol in order to avoid potential side effects of corticosteroids and intravenous vincristine. The patient tolerated oral propranolol well. The dose was gradually increased over three days to 2 mg/kg/day given every 8 hours with close monitoring of blood pressure, heart rate, and premeal blood glucose levels. Her platelet counts increased without the need for platelet transfusion. The sequential platelet counts are shown in Figure 2. The lesion became softer and paler after the fifth day of propranolol. In 10 days, the KMP resolved with normalization of platelet count and coagulation profiles without evidence of relapse. The lesions also regressed in size, as shown in Figure 1. Plasma D-dimer and fibrinogen levels returned to normal in one week.

After eight months of follow-up, the patient still had stable cutaneous lesions while receiving propranolol monotherapy. Propranolol was then discontinued with regular hematologic monitoring in order to detect late relapse of the disease. No adverse effects were observed in the patient. Up to date, she has had hematologic remission for six months after discontinuation of propranolol. The primary cutaneous lesion has become a pale pink, 1 cm sized skin lesion.

## 3. Discussion

KMP is associated with significant morbidity and mortality caused by its refractoriness to treatment and bleeding complication, a condition which requires aggressive treatment. In our patient, a diagnosis of KMP was done based on clinical findings of vascular mass, elevated serum D-dimer, and thrombocytopenia without histopathologic diagnosis. These correlated with consensus-derived practice standards plan for complicated kaposiform hemangioendothelioma [10] reported in 2013; 64% of experts' centers did not think that a tissue biopsy was necessary to confirm diagnosis of KMP. But the consensus recommended magnetic resonance imaging (MRI) as the gold standard imaging test for KMP; we used CT scan instead because of the availability and risks of anesthetic procedures in this very young age of patient. The interventions, including total resection and intravascular embolization, are frequently incapable of being done in KMP because of bleeding complications and anatomical sites of KMP. Corticosteroids and vincristine have been considered the first-line treatment for KMP [6, 8, 11, 12]. In recent studies, corticosteroids for KMP show variable responses (20–70%), depending on the dosage [12, 13], and can cause several adverse side effects such as severe hypertension, growth restriction, and osteoporosis [13]. Vincristine is usually used in combination with other therapies for severe, life-threatening cases because of its good tolerance and response. Several studies used vincristine monotherapy for KMP with good response rates of 86–100% [8, 11], but this agent has not been used widely because of its neurotoxicity and other side effects [8, 12, 14].

In 2008, Léauté-Labrèze et al. [15] reported the effectiveness of propranolol in treatment of IHs but with an unclear mechanism of action. Since then, propranolol has been recommended as a first-line treatment of IHs [6] but not for KHE or KMP. Only a few literature reviews, pertaining to the role of propranolol when used in addition to a steroid or the vincristine treatment for KMP, reported both good [8, 16] and poor responses [13, 17].

Recently, Drolet et al. [10] reported consensus-derived practice guidelines for complicated KHE and recommended corticosteroids and intravenous vincristine as a frontline treatment for KHE with KMP. Our patient already had had complete remission clinically and hematologically before this consensus was published. With propranolol monotherapy for our patient, the gradual response to the treatment was closely observed during a ten-day period with normalization of hematologic parameters. This effectiveness of treatment might be the result of the small initial size of the primary tumor and the mild severity of coagulopathy. As mentioned above, our patient had an initial tumor size of 5 cm × 5 cm and platelet count of 55,600/*μ*L before starting treatment. These results may be compared to the study of Chiu et al. in 2012 [9], which reported the variable responses of propranolol monotherapy for three patients with KMP (two of the patients did not respond to treatment, but one patient with a 5 cm tumor had a complete response). Primary vascular tumors less than 5-6 cm in size might, therefore, respond better than a larger one to propranolol treatment. The severity of coagulopathy, including degree of thrombocytopenia, plasma D-dimer, and fibrinogen levels, might also determine responsiveness to treatment. Most patients with KMP with no or a partial response to propranolol in the literature had severe thrombocytopenia (<20,000/*μ*L) and markedly elevated D-dimer levels [8, 13, 17].

As most patients with KMP have severe thrombocytopenia and coagulopathy, it remains unclear how aggressive the therapy should be for patients who have mild KMP. While the consensus guidelines recommend vincristine and corticosteroids, we demonstrated the efficacy and feasibility of propranolol monotherapy (2 mg/kg/day in three divided doses) for mild KMP with good response in both hematologic parameters (platelet counts, plasma D-dimer, and fibrinogen levels) and induction of tumor regression. Long-term follow-up and durability of response are unknown at this time. In severe KMP, the standard treatment with corticosteroids, vincristine, and/or intervention should be administered without delay.
